# Surface recombination and charged exciton in nanocrystal quantum dots on photonic crystals under two-photon excitation

**DOI:** 10.1038/srep05039

**Published:** 2014-06-06

**Authors:** Xingsheng Xu

**Affiliations:** 1 State Key Laboratory of Integrated Optoelectronics, Institute of Semiconductors, Chinese Academy of Sciences, Beijing 100083, China

## Abstract

In this study, the two-photon excited fluorescence spectra from cadmium selenide quantum dots (QDs) on a silicon nitride photonic crystal (PhC) membrane under femtosecond laser irradiation were investigated. These spectra can be fit to a tri-Gaussian function in which one component is negative in amplitude, and in which the Gaussian components with positive amplitude are assigned to exciton emission and charged-exciton emission and that with negative amplitude is assigned to absorption from surface recombination. The photonic crystal enhance the charged-exciton emission and exciton emission and, at the same time, also the absorption from surface recombination. Both the charged-exciton emission and the surface recombination are related to Auger recombination; therefore, the photonic crystal controls both radiative recombination and non-radiative recombination. The asymmetries of the two-photon excited fluorescence spectra are due to not only the location of the resonant guide mode of the PhC slab but also the enhancement of the absorption from surface recombination by PhC.

Colloidal quantum dots (QDs) are important materials in optoelectronics and have novel applications as light sources^1^, detectors^2^, photovoltaic^3^ and bio-photonic devices^4^. However, they suffer from a major disadvantage, namely photoluminescence (PL) intermittency, or ‘blinking'. Many papers have been published on this topic, and several mechanistic models have been proposed for the luminescence blinking of colloidal QDs^5^. In the early-charging model^6^, the *off* state is assigned to an ionised state of the QD formed by the trapping of a photo-excited electron. The *off* state occurs when a hole is localised in the QD core and an electron is ejected to a nearby acceptor or trap site on the surface or in the surrounding matrix. A significant challenge to this model is the demonstration that Auger decay alone is insufficient to create a dark state in a singly charged QD. The low-QY dark state also indicates that the charging model is of limited utility in explaining the photo physics of PL intermittency^7^.

In the model for PL intermittency involving multiple recombination centres^8^, the blinking is due to the activation and deactivation of trapping states at the surface or in the core/shell interface of the QD. In the model involving a combined mechanism of surface recombination and Auger recombination^9^,^10^, the *off* state is a non-radiative state due to Auger recombination or surface recombination, and blinking is suppressed when the surface trapped state is filled by external electrons. From this brief description of these models for PL intermittency in QDs, it is clear that Auger recombination and trapped states are important factors involved in the blinking phenomenon. Because the surface state is one of the trapped states^11^, a number of important questions need to be answered to determine the available mechanism of PL intermittency^11^. For example, how does the Auger process and surface recombination affect the radiative properties of colloidal QDs? The actual physical mechanism of photoluminescence intermittence is still actively debated.

Michele Saba *et al.* controlled the accumulation of charges and the activation of charge traps. They found that both the photo-charge and charge trapping contribute to photoluminescence quenching, and both processes can be reversibly induced by light. Moreover, charge accumulation and trap formation are strongly sensitive to the environment^12^. Current obstacles to improving the performance of quantum-dot light-emitting diodes include various factors, such as nonradiative recombination at surface defects, multicarrier Auger recombination or electron-hole separation due to the applied electric field. In core/shell QDs, electrons and holes are partially separated in space, which results in Coulombic repulsion in the case of multicarrier states and leads to a blue shift of the corresponding emission band. In a QD-LED, competing effects from heating and Coulombic repulsion coexist^13^.

In this study, two-photon excited fluorescence (TPF) spectra from cadmium selenide (CdSe) QDs on a silicon nitride (SiN) photonic crystal (PhC) membrane are studied. Results show that the spectrum is significantly asymmetric with PhCs under different excitation intensities. In addition, the fraction and the centre wavelength of the absorption and the charged-exciton emission change with the lattice constant but are nearly constant with varying pumping power. The asymmetries in the TPF spectra are partly due to the location of the resonant guide mode of the PhC slab and partly due to PhC enhancement of the absorption from surface recombination. An analytical method is proposed in which the asymmetric PL spectrum from QDs is fit to a multi-Gaussian function with one negative amplitude, which is attributed to photo-induced loss. It is revealed that the spectrum can be fit to a tri-Gaussian function, in which the components with positive amplitude represent exciton emission and charged exciton emission and the component with negative amplitude represents photo-induced loss, which may mainly be due to absorption from surface recombination^14^,^15^,^16^ assisted by an Auger process. From the fitting parameters, it is possible to describe the QD microscopic carrier relaxation and non-radiative processes.

## Results

**Figure 1:**
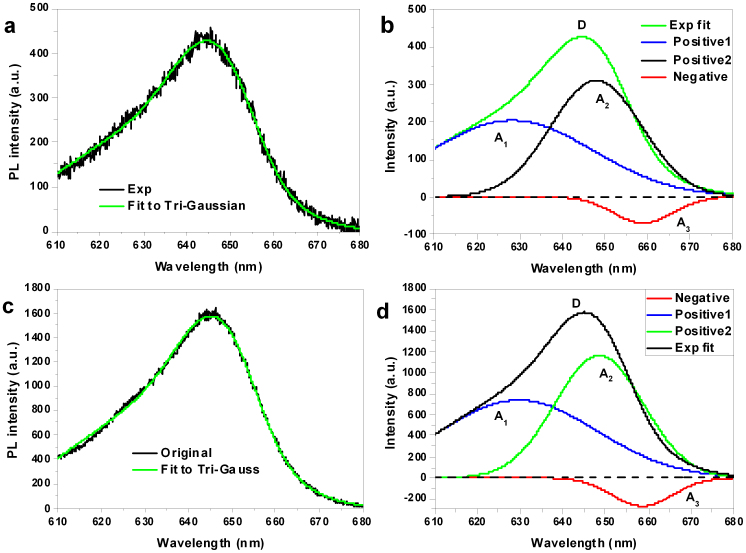
Recorded and fitted two-photon excited fluorescence (TPF) spectra of QDs on a PhC with a lattice constant 560 nm. (a), TPF spectrum fitted to a tri-Gaussian function with one negative amplitude under an excitation power of 4.64 mW. (b), The three fitted Gaussian components are shown separately; curves A_1_ and A_2_ correspond to the components with a positive amplitude, and curve A_3_ corresponds to the component with a negative amplitude. The curves are shown together with their superposition (curve D) and the measured spectrum. (c), QD TPF spectrum fit to a tri-Gaussian function with one negative amplitude under an excitation power of 9.3 mW. (d), The three fitted Gaussian components in c are shown separately; curves A_1_ and A_2_ correspond to the components with a positive amplitude, and curve A_3_ corresponds to the component with a negative amplitude. The spectra are shown together with their superposition (curve D) and the measured spectrum.

The experimental setup and material preparation are described in the Methods section. The recorded TPF spectra of 655 ITK QDs (Invitrogen Inc.) on a PhC with a lattice constant of 560 nm and a radius of 210 nm with excitation powers of 4.64 mW and 9.3 mW (after objective) are shown in Fig. 1. On the PhC, the entire TPF spectrum of the QDs differs from that on a SiN membrane without the PhC (Supplementary Fig. S1). It can be observed that the spectrum is very asymmetric, because the PL intensity at shorter wavelengths is enhanced considerably by the PhC.

To investigate the physical mechanism underlying this asymmetry in the PL spectrum, the measured spectrum is fit to a tri-Gaussian function 
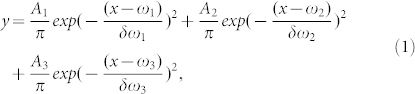
where A_1_, A_2_, and A_3_ are the amplitudes of the three Gaussian components; δ*ω*_1_, δ*ω*_2_ and δ*ω_3_* are the spectral widths; and *ω*_1_, *ω_2_* and *ω_3_* are the centre wavelengths.

In the first fit, shown in Fig. 1a, both amplitudes A_1_ and A_2_ are positive, while A_3_ is negative. In this fit, the Gaussian components in equation (1) have amplitudes of *A*_1_ = 204.3 with a centre wavelength ω_1_ = 628.2 nm, *A*_2_ = 310.9 with a centre wavelength ω_2_ = 648 nm, and A_3_ = −70 with a centre wavelength ω_3_ = 659 nm. The long-wavelength component is conspicuous for its negative amplitude, which is attributed to photo-induced loss (including absorption from surface recombination and photo-bleaching). The components with positive amplitudes represent radiation from the QDs. Thus, the TPF curve is composed of three components: two emission bands at approximately 648 nm and 628.2 nm, corresponding to the centre wavelength of the exciton emission and the charged-exciton emission, respectively, and a band at approximately 659 nm, corresponding to the photo-induced loss due to absorption by surface recombination^15^,^16^ or photo-bleaching, both of which are related to the surface states. Thus, the band at approximately 659 nm represents the location of the energy level of the surface state. The spectral width reaches 8.081 meV (10.02 nm), which means that there are multiple surface states^8^.

To clearly understand the processes of emission and loss, the respective curves for the fitted Gaussian components with negative and positive amplitudes are shown separately in Fig. 1b. The superposition of curves A_1_, A_2_ and A_3_ (the Gaussian components with positive, positive, and negative amplitudes, respectively) results in the tri-Gaussian curve D, which fits well with the original experimental data (Exp). Curves A_1_ and A_2_ represent the emission and curve A_3_ represents the photo-induced loss. It can be observed that there is a loss band towards longer wavelengths and that the PL intensity is enhanced towards shorter wavelengths; thus, the PL spectrum is very asymmetric. The loss band is located at the longer wavelength spectrum band, where the band is not broad. Compared with the spectrum of the QDs on SiN without PhC (Supplementary Fig. S1), the spectrum in the shorter-wavelength range is much broader. It is obvious that so broad a spectrum cannot arise from band-edge exciton emission alone. The positive bands of TPF spectrum on PhCs shift to blue, which may come from both the charged exciton (trion) emission and from enhancement by PhC, where the enhancement by the PhC may affect both the charged-exciton emission and the exciton emission.

The spectrum of the QDs on the same PhC under an excitation power of 9.3 mW and its fit to a tri-Gaussian function are shown in Fig. 1c, and the three Gaussian components are shown separately in Fig. 1d, including curves A_1_ and A_2_ for the components with positive amplitude and curve A_3_ for the component with negative amplitude together with their superposition (curve D) and the measured spectrum. The superposition, curve D, fits well to the measured spectrum (curve Exp). Of the three Gaussian components, that with a negative amplitude and a centre wavelength at 658.6 nm (curve A_3_) is similar to the negative-amplitude component under 4.64 mW in Fig. 1b and represents photo-induced loss. The component with a centre wavelength at 648.5 nm (curve A_2_) represents exciton emission, and the component with a centre wavelength at 629.7 nm (curve A_1_) represents positive trion emission^12^.

Under light irradiation, charged exciton generation and charged-exciton emission (shorter-wavelength components of the tri-Gaussian fitting) will be enhanced as a result of coupling to the guide-resonant mode^17^ in the PhC waveguide slab. Because of the limited response speed of the silicon CCD used in the observations, the measured spectrum at shorter wavelengths is mainly from charged-exciton emission or from a state-filling effect rather than from multi-excitons. The ratios of the amplitude of the charged-exciton emission to that of the exciton emission can be calculated, and it is found that the ratio of the amplitude of the charged exciton to that of the excitons is 63.6% (A_1_/A_2_) for the PhC with a lattice constant a = 560 nm. In the charging model for the blinking of single CdSe/ZnS nanocrystals, charged-exciton emission corresponds to the *off* state and exciton emission to the *on* state^6^. However, we obtained a ratio of the emission of *off* state (emission of trion) to that of the exciton *on* state exceeding 60%, and it seems that the *off* state is too bright. This phenomenon indicates that the charging model for PL blinking is inadequate^18^.

Measurements of the QD lifetimes on a SiN film (Supplementary Fig. S2) confirmed the above result. Under light irradiation, ionization and charge separation occur, and charged excitons will be generated after carrier recombination. High excitation intensities lead to Auger-assisted multi-exciton and charged exciton generation. We measured the lifetime of the QD PL under different excitation powers and found that the lifetime can be fit to a tri-exponential (three-term exponential) function (Supplementary Fig. S2, and Table S1), with the ratios of the three components varying with the excitation power. The spectral width is related to the PL lifetime of the QDs, and the variation tendency of the spectral width with excitation power is in accordance with that of the lifetime.

**Figure 2:**
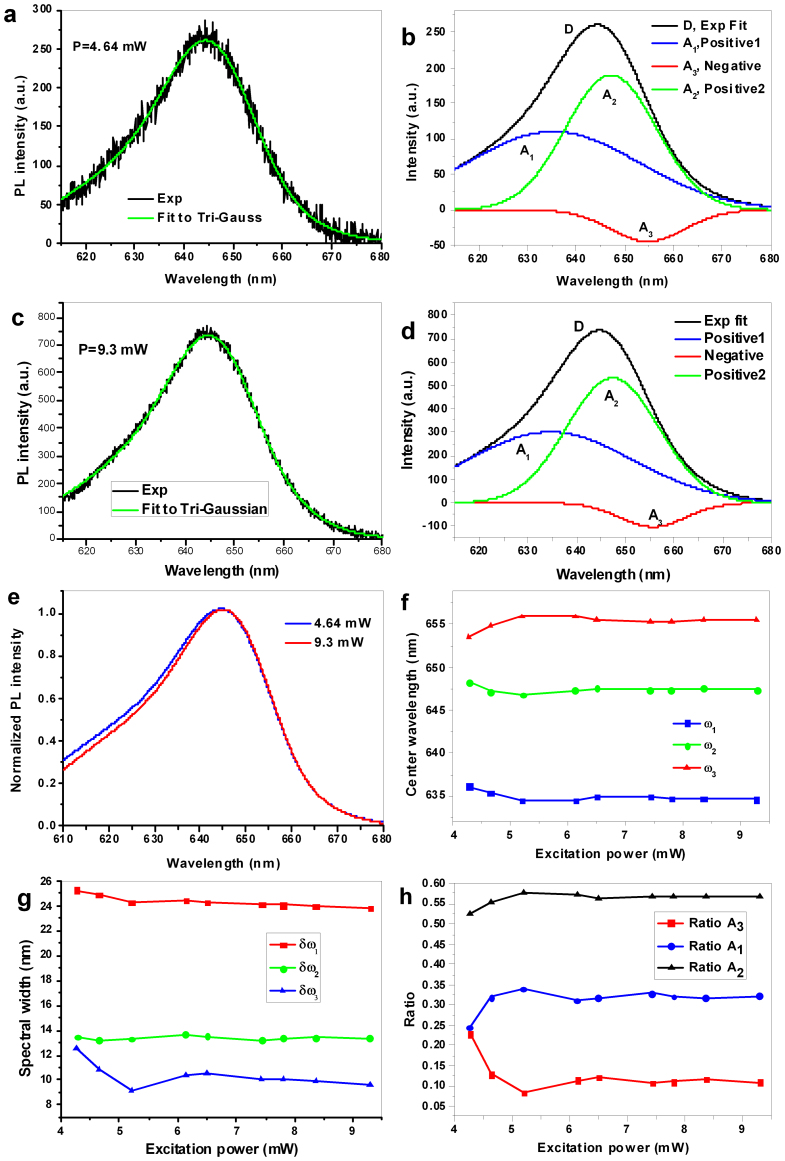
TPF spectrum and Gaussian fitting for QDs on a PhC with a lattice constant of 580 nm. (a), TPF spectrum fitted to a tri-Gaussian function with one negative amplitude under an excitation power of 4.64 mW. (b), The three fitted Gaussian components are shown separately; curves A_1_ and A_2_ correspond to the components with a positive amplitude, and curve A_3_ corresponds to the component with a negative amplitude. The spectra are shown together with their superposition (curve D) and the measured spectrum. (c), TPF spectrum fitted to a tri-Gaussian function with one negative amplitude under an excitation power of 9.3 mW. (d), The three fitted Gaussian components in (c) are shown separately; curves A_1_ and A_2_ correspond to the components with a positive amplitude, and curve A_3_ corresponds to the component with a negative amplitude. The spectra are shown together with their superposition (curve D) and the measured spectrum. (e), Comparison of the fitted spectra to the tri-Gaussian under the excitation powers of 4.64 mW and 9.3 mW. (f–h), Fitting parameters for the tri-Gaussian function for different excitation intensities (subscripts ‘1', ‘2' and ‘3' indicate the long-, middle- and short-wavelength components, respectively): (f), centre wavelengths *ω*_1_, *ω*_2_ and *ω_3_*; (g), spectral widths δ*ω*_1_, δ*ω*_2_ and δ*ω_3_*; (h), ratios of components, A_1_/(A_1_ + A_2_ + A_3_), A_2_/(A_1_ + A_2_ + A_3_) and A_3_/(A_1_ + A_2_ + A_3_).

To analyse the photo-physics of the asymmetric spectrum, TPF spectra under different excitation intensities were investigated (see Methods). TPF spectra of the QDs on the PhC with a lattice constant of 580 nm under two excitation powers fit to a tri-Gaussian function are shown in Fig. 2a and c. The spectra can be fit well to a tri-Gaussian function with one negative amplitude. The spectrum on the PhC is blue shifted compared with the spectrum on SiN without a PhC. Furthermore, the spectrum at higher excitation intensities is red shifted compared with that at lower excitation intensities in the range of low excitation power (Fig. 2e). The centre wavelengths, the spectral widths and the ratios of the components for the tri-Gaussian fit are shown as functions of the excitation intensity in Fig. 2f, g and h, respectively. It can be observed in Fig. 2f that the centre wavelength of the long-wavelength component (assigned to photo-induced loss) decreases, whereas those of the shorter-wavelengths components increase, with increasing excitation power from 4.17 mW to 5.17 mW. In addition, when the excitation power is increased from 5.5 mW to 9.3 mW, the centre wavelength of both the positive amplitude and the negative amplitude remains nearly constant. As the excitation intensity decreases towards its initial value, the centre wavelength of the exciton also decreases towards its initial value (not shown). In Fig. 2h, in the higher power range (5.5–9.3 mW), the ratio of the middle-wavelength component (exciton emission) and that of the long-wavelength component (absorption) remain almost constant; the average ratios of the shorter-, middle- and longer-wavelength components are 0.319, 0.568 and 0.112, respectively. With increasing pump power in the low-power range, the absorption spectrum shifts to red, whereas that of the exciton emission shifts to blue, and the ratio of the component of the absorption due to surface recombination increases, whereas that of exciton (including charged exciton) emission decreases. The spectral widths of both the exciton emission and the photo-induced loss display almost no changes with increasing excitation power in the higher power range (Fig. 2g). It was found that the changes in the spectral widths and centre wavelengths of both the long- and short-wavelength components could be reversed by changing the excitation intensity (not shown), which demonstrates that the change in the spectrum is not due to a change in the size of the QD. With changing excitation intensity in the range from 4.17 mW to 5.17 mW, the ratios of the absorption and the exciton emission show opposite trends, as do the centre wavelengths of these components, while in the higher-power range, the characteristics of the charged exciton and exciton emission and the photo-induced loss are nearly insensitive to the excitation power, which appears to be consistent with the charging model for the blinking of QDs, where the dark state is independent from the excitation power. The long-wavelength component mainly represents absorption from Auger-assisted surface recombination. In the experiments reported in Ref. [19], at high pump intensities, below the main bleaching band, a photo-induced absorption band arose at a wavelength longer than that of the photoluminescence band owing to the activation of a new hole relaxation path^19^. In our case, the surface recombination state may behave similarly to this hole relaxation path, trapping holes from the valence band and absorbing corresponding light energy.

We measured and analysed many spectra from QDs on PhCs with different lattice constants (Fig. S3 for examples) and found that the intensities at shorter wavelengths for all the spectra from the different PhCs were enhanced compared with those at longer wavelengths. This asymmetric enhancement is not only due to the location of the resonant guide mode of the PhC slab. By calculating the photonic band structure of the PhC, it can be found that the PhC slab also has resonant guide modes in the longer-wavelength region, which are similar to those in the short wavelength region. Under light irradiation, active surface trap states are present and surface recombination occurs. During recombination, the surface state traps carriers from the valence band^20^, which then needs to absorb light of the corresponding energy. Due to potential barrier at a QD boundary (see Fig. 4), this process will absorb radiation from the band-edge exciton emission. However, if an electron in a higher excited state (a ‘hot carrier') is transferred to the surface state, then there is no need for photon absorption.

**Figure 3:**
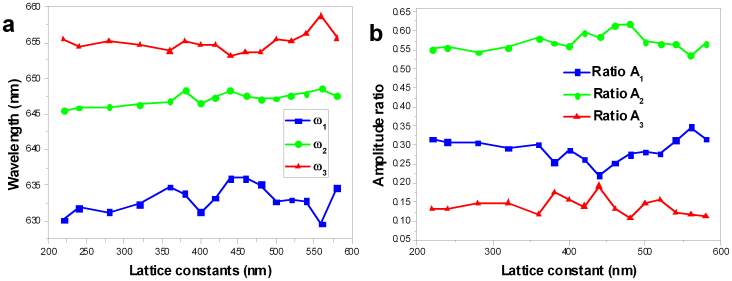
Fitting parameters for the tri-Gaussian function as a function of the PhC lattice constant. (a), Centre wavelengths. (b), Ratios of the components.

Spectra under an excitation power of 9.3 mW on PhCs with different lattice constants were collected and fit to tri-Gaussian functions with one negative amplitude. The centre wavelengths and the ratios of the long-, middle- and short-wavelength components are shown as functions of the lattice constant in Fig. 3a and b. The long-wavelength component corresponds to photo-induced loss, the middle-wavelength to exciton emission and the short-wavelength component to charged-exciton emission. For different lattice constants, the smaller the ratio of the component of the charged-exciton emission is, the longer its centre wavelength is. On the same PhC, the trend of variation of charged-exciton emission (including the ratio of the amplitude and the centre wavelength) displays opposite behaviour to that of photo-induced loss. It is difficult to determine a regular relationship between the PL intensity and the spectral width and between the PL intensity and the centre wavelength. The average ratios of the shorter-, middle- and longer-wavelength components for the PhCs under an excitation power of 9.3 mW are 0.286, 0.575 and 0.139, respectively, while those on SiN without PhCs are 0.321, 0.638, and 0.040, respectively (Fig. S1). The results of the ratio of surface recombination are in accord with those reported by Sewall et al.^15^, where the fraction of carriers at the surface was approximately 0.35–0.1 for CdSe QDs ranging from 1.5–3.4 nm in radius.

## Discussion

Compared with the spectrum of the QDs on SiN without PhC, the centre wavelength of the spectrum on a PhC is blue shifted as a result of charged-exciton emission. As described above, the total spectrum on a PhC is red shifted with increasing pump power due to heating effect or to negative trion emission^21^. Simultaneously, the centre wavelength of photo-induced loss shifts to blue with increasing pump power. This indicates that the surface recombination state (photo-induced loss) first absorbs photons with relative longer wavelength, that is, the relatively low-energy photons, and then shifts to higher-energy photons with increasing power. This phenomenon is similar to the state-filling effect^22^ related to photobleaching^25^. The absorbed photons first excite a carrier to a relatively low-energy level of the surface state, and after the lower energy levels of the surface state have been filled, the process moves to higher energy levels. Therefore, the fitted Gaussian component with a negative amplitude at longer wavelengths represents the loss of photons, where the wavelength of this longer-wavelength component is centred on the energy level of the surface states.

**Figure 4:**
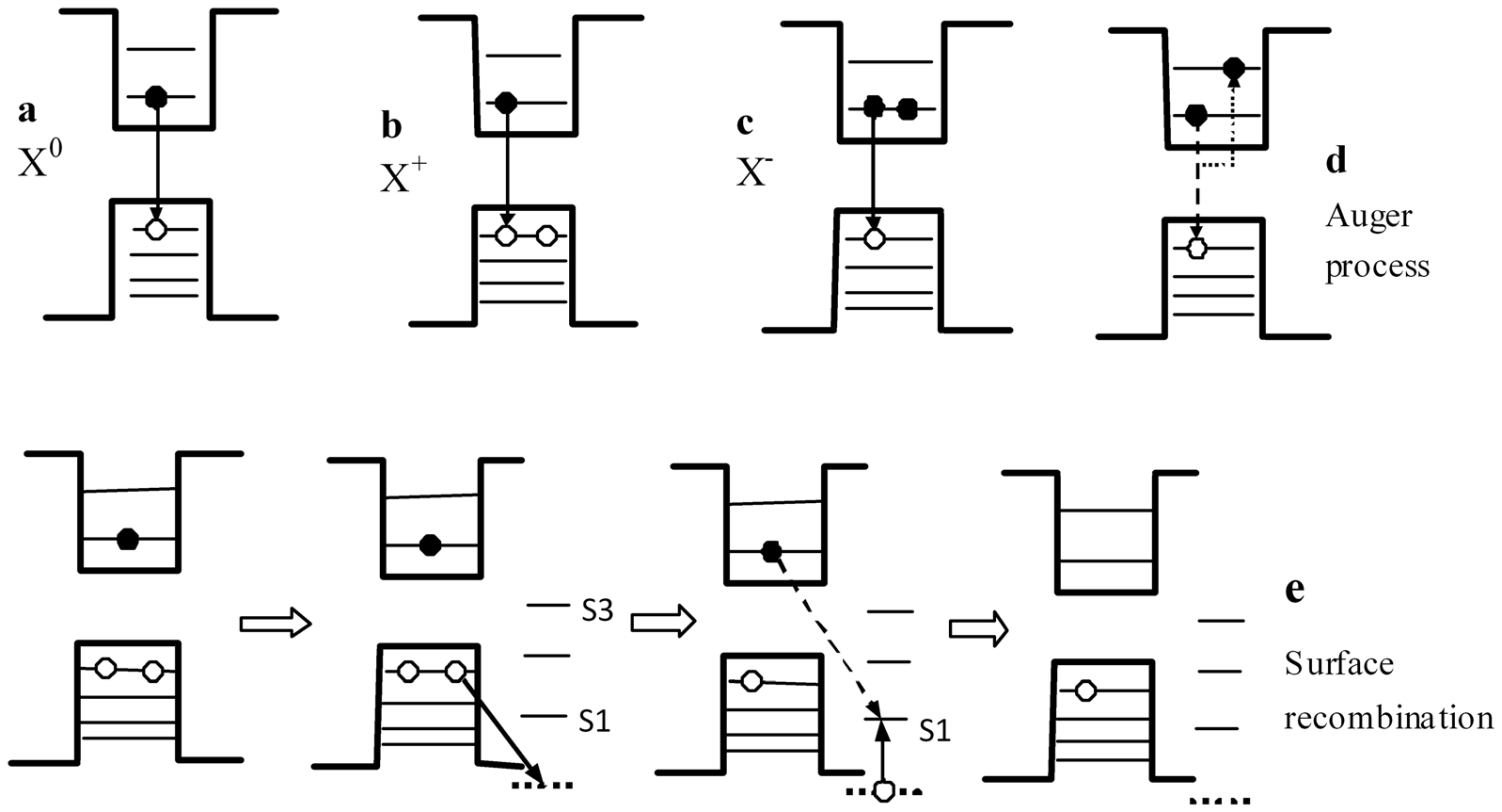
Schematic diagram of the band structure of the QDs illustrating carrier trapping and optical transitions. (a), exciton emission X^0^; (b), charged exciton (positive trion) emission X^+^; (c), negative trion emission X^−^; (d), Auger recombination; (e), photo-induced loss of the emission light due to surface recombination, in which the hole is driven to the surface state, and surface recombination takes place. The horizontal lines besides band gap represent the surface states, the horizontal dotted line represents the potential of QD boundary.

A schematic diagram in Figure 4 describes carrier trapping and optical transitions in QDs, through which it is possible to summarise the carrier relaxation and recombination processes, including radiative and non-radiative processes. These include (a) exciton emission, where an electron transfers from conductive band to valence band; (b) positive charged exciton (positive trion) emission; (c) negative trion emission^21^; (d) Auger recombination; and (e) photo-induced loss of the emitted light due to surface recombination, in which the carrier is driven to the surface state, this process will absorb light to trap hole to surface state S1 due to the potential barrier at a QD boundary, which may be assisted by Auger process.

Under irradiation with the excitation laser, the QD is ionised and the electron is ejected out the QD, leaving a positive QD core. Fig. 4(b) represents a positive charged QD. As a result of charging, the energy level will split under coulomb interactions, causing the centre wavelength of the spectrum of the positive charged exciton to shift to the blue relative to that of the exciton. Under the action of the electrical field generated by the charge separation between the QD core and the surface, which may introduce a dc Stark effect^19^,^23^, charged-exciton emission (Fig. 4(b) and (c)) occurs. At high pump intensities, the absorption spectra showed a derivative-like feature that was a clear signature of a dc Stark effect^19^. Similar transient absorption features have also been reported in QDs in the case of nonlinearities arising from charge separation induced by carrier trapping^19^ and the bi-exciton effect^24^.

Irradiation by the pump light will activate trap states, which in QDs are mainly surface states. A trap state can trap a hot electron (from a higher excited state)^23^. As a result of hole trapping, new trapped states are generated via the dc electric field^20^. Because the new hole relaxation channel is associated with efficient charge separation, the states activated by high pump intensities that trap holes are most likely surface/interface states^19^. This surface-state model is in accord with the multiple-recombination-centres model^8^ and the trap-state distribution described in Refs [7,8,23]. According to the prediction of the charging model, the exponent in the power law and thus the energy level of the trap state are independent of the excitation power, which is also consistent with the results we obtained from the QD spectrum; namely, that the ratio of the loss due to surface recombination maintains almost constant in higher excitation power range (Fig. 3(h)). The power dependence in lower pump power range and the broad spectral width of the photo-induced loss spectrum indicate that the surface trap states are multiple trap states. Our results are in accord with the explanation in the multiple-trap model for the blinking of single QDs^25^; owing to the static distribution of trapping and de-trapping rates, which vary with trapping distance, power-law *off*-time distributions are naturally obtained^25^.

The QD spectrum on SiN without a PhC under high excitation intensity also shows some asymmetry (Fig. S1). PhCs enhance the asymmetry of the TPF spectrum (Fig. 1, 2). As mentioned above, the asymmetric enhancement is due not only to the location of the resonant guide mode of the PhC slab but also to photo-induced loss, such as the absorption of recombination in trapping states, especially surface states. This research shows that the asymmetric spectrum of TPF can be fit to a tri-Gaussian function with one negative amplitude component located in the longer-wavelength region of the spectrum, where the negative amplitude represents absorption due to photo-induced loss. The high degree of asymmetry in the spectrum arises for two reasons: first, the PhC enhances the charged-exciton emission and exciton emission; second, there is a loss, or absorption band, located in the longer-wavelength region. This photo-induced loss (photo-bleaching or absorption) is due to surface recombination assisted by an Auger process. Under laser irradiation, the surface states will trap holes, leading to absorption of the light emitted by the QD. With further increases in excitation power, the lower energy levels are filled, and holes are pumped into the higher energy levels of the surface states. Meanwhile, the surface states also trap electrons from the excited state (hot carriers), with electron transferring to the surface states very rapidly. The trapping of carriers in the surface states is followed by non-radiative surface recombination. As Sippel *et al.* reported, two cases can occur: in one, an electron relaxes to the ground state by an Auger-like process; in the other, a hole is trapped by a surface state, impeding the Auger-like process^26^.

In conclusion, we studied the two-photon excited fluorescence spectra from cadmium selenide (CdSe) QDs on silicon nitride (SiN) photonic crystal (PhC) membranes. The uses of PhCs and curve-fitting to a tri-Gaussian function with negative amplitude are two important methods for analysing the PL spectrum of QDs. The component of the negative amplitude of the tri-Gaussian function is attributed to absorption due to surface recombination or photo-induced loss by photo-bleaching, while the components with positive amplitudes are attributed to charged exciton and exciton emission. The absorption process due to surface recombination is assisted by Auger recombination. The average ratios of the components of the charged exciton, exciton emissions and the photo-induced loss on PhCs are 0.286, 0.575 and 0.139, respectively. The average ratio of photo-induced loss on PhCs is several times larger than that without PhCs. From the spectra of QDs on PhCs, the charged-exciton emission and surface recombination can be investigated together.

## Methods

The colloidal QDs were Qdot 655 ITK (Invitrogen Inc.), with an emission centre wavelength at 655 nm. They were cast onto the surfaces of SiN films with two-dimensional PhCs, which were fabricated by electron-beam lithography and reactive-ion dry etching. The PhC lattice constants were ranged from 580 nm to 220 nm with interval of 20 nm. The sample was excited by a femtosecond laser with repetition frequency 80 MHz and wavelength 800 nm focused by a 40× objective lens, and the TPF from the QDs below 800 nm was fed to a monochromator with a silicon CCD. The measurement system collected the signal continuously and simultaneously with continually varying the excitation power. At every power there was a dwell time of a few seconds while the TPF spectra from the QDs were recorded. Two short-pass filters (at band-edge wavelengths of 800 nm and 720 nm) were inserted before the spectrometer. The excitation power was controlled continuously by a Glan prism and a *λ*/2 plate, and the spectra under different pump powers were recorded simultaneously by a monochromator with a silicon CCD. The excitation power was increased from the minimum to the maximum and then decreased from the maximum to the minimum under continuous control by a Glan prism and a *λ*/2 plate.

The fitting of the spectra was performed using Matlab (version R2009a).

## Supplementary Material

Supplementary Information Suppplemtary materials
