# Supplementary material for: Surface recombination and charged exciton in nanocrystal quantum dots on photonic crystals under two-photon excitation
Source: Sci Rep. 2014 Jun 6;4:5039. doi: 10.1038/srep05039 (PMC4047539; doi:10.1038/srep05039)
Supplement: Supplementary Information — Suppplemtary materials [file srep05039-s1.doc]

**Supplementary information**

**Surface recombination and charged exciton in nanocrystal quantum dots on photonic crystals under two-photon excitation**

**Xingsheng Xu**

State Key Laboratory of Integrated Optoelectronics, Institute of Semiconductors, Chinese Academy of Sciences, Beijing 100083, China. E-mail: [xsxu@semi.ac.cn](mailto:xsxu@semi.ac.cn)

For comparison with the results in the main text, two-photon excited fluorescence (TPF) spectra of QDs on SiN without a photonic crystal (PhC) under excitation powers of 6.5 mW and 9.3 mW are shown in Fig. S1a, b. At the low excitation intensity (Fig. S1a), the spectrum can be fit relatively well to a single Gaussian function, while at the high excitation intensity (Fig. S1b), the fit to a single Gaussian function is poor, and the spectrum can instead be fit to a tri-Gaussian function, in which the amplitude of the longer-wavelength component is negative and those of the two shorter-wavelength components are positive. The centre wavelengths of the components are shown in Fig. S1c. It can be observed that with increasing excitation power, ω_3_ shifts to a value of approximately 656 nm, while ω_1_ is approximately 641 nm, and ω_2_ is approximately 648 nm. Figure S1d shows that the ratio of the longer-wavelength component is less than 0.1 (averaged one 0.04), which indicates that the QDs on SiN without PhC do not undergo such strong surface recombination as they do in the presence of a PhC. The average ratio of the charged exciton is approximately 0.32, which is similar to that on a PhC with a lattice constant of 580 nm, while the ratio of the band edge emission is higher than that on a PhC.

**Supplementary Figure S1 | Recorded and fitted TPF spectra under different excitation powers.** **a,** 6.5 mW. **b,** 9.3 mW. **c, d,** Fitting parameters for the TPF spectrum on a SiN membrane without PhC (subscripts ‘1’, ‘2’ and ‘3’ indicate the long-, middle- and short-wavelength components, respectively): **c,** variation of the centre wavelengths ω_1_, ω_2_ and ω_3_ with excitation power; **d,** variation of the ratio of the components A_1_/(A_1_+A_2_+A_3_), A_2_/(A_1_+A_2_+A_3_) and A_3_/(A_1_+A_2_+A_3_) with excitation power.

**Supplementary Figure S2 | Decay process of photoluminescence from QDs on a SiN film with a PhC.** The green and red curves are fits to tri-exponential functions.

Measurements of the emission lifetime from the QDs on a SiN film with a PhC are shown in Fig. S2. On PhCs, if the emission is in the range of the photonic band, the radiation rate will not change considerably compared with that without PhCs, being approximately 0.8 times the value for the material without PhCs^1^. For PhCs with lattice constants of 580 nm and 560 nm, the emission is located in the photonic band, and therefore the lifetime of the emission from the QDs does not change significantly and is determined by the radiation process of the QDs. The decay process can be fit to a tri-exponential function, that is, the sum of three exponential functions. The fitting parameters are listed in Table S1. For a lattice constant of 560 nm, the lifetime of the fast component, which represents multi-exciton emission, is 430 ps; the lifetime of the slow component, which represents exciton emission, is 19.93 ns; and the lifetime of the middle component, which represents trion emission, is 5.52 ns. The trion lifetimes are similar to those obtained in Ref. [3]. The ratio of the amplitude of the trion component to the total amplitude is 38.8%. It can be observed from Table S1 that for a PhC with other lattice constants, this ratio is also greater than 30%, reaching 43% for a lattice constant of 360 nm.

**Supplementary Table S1** Parameters of the decay processes fit to a tri-exponential function, where A_1_, A_2_, and A_3_ are the amplitudes and t_1_, t_2_, and t_3_ are the time constants for the three components of the tri-exponential, and *A*_t_ is the total amplitude.

| PhC lattice constant *a* (nm) | *A*_1_ | *t*_1_  (ns) | *A*_2_ | *t*_2_  (ns) | *A*_3_ | *t*_3_  (ns) | *A*_t_ | *A*_2_/*A*_t_ (%) |
| --- | --- | --- | --- | --- | --- | --- | --- | --- |
| 580 | 2,894 | 20.38 | 1,966 | 5.74 | 246 | 0.41 | 5,106 | 38.5 |
| 560 | 2,373 | 19.93 | 1,668 | 5.52 | 262 | 0.43 | 4,303 | 38.8 |
| 480 | 6,873 | 12.54 | 3,614 | 2.85 | 644 | 0.22 | 11,131 | 32.5 |
| 360 | 13,171 | 18.75 | 12,695 | 4.58 | 3,610 | 0.40 | 29,476 | 43.1 |
| SiN film | 981 | 19.39 | 623 | 6.34 |  |  | 1,604 | 38.8 |

**Figure S3 TPF spectrum and Gaussian fitting for QDs on a PhC with a lattice constant of 360 nm.** **a**, TPF spectrum fit to a tri-Gaussian function under an excitation power of 4.65 mW. **b**, TPF spectrum fit to a tri-Gaussian function under an excitation power of 9.3 mW. **c–f,** Fitting parameters for the tri-Gaussian function under different excitation intensities (subscripts ‘1’, ‘2’ and ‘3’ indicate the long-, middle- and short-wavelength components, respectively): **c,** amplitudes for the three fitted components varying with excitation power; **d,** variation of the ratio of the components A_1_/(A_1_+A_2_+A_3_), A_2_/(A_1_+A_2_+A_3_) and A_3_/(A_1_+A_2_+A_3_) with excitation power; **e,** variation of the centre wavelengths ω_1_, ω_2_ and ω_3_ with excitation power; **f,** spectral widths of the three components.

The TPF spectrum from the QDs on a PhC with a lattice constant of 360 nm and the corresponding fit to a tri-Gaussian function is shown in Fig. S3a, b. As observed in Fig. S3c, the amplitudes of the three components increase with increasing excitation power, reaching saturation near the high excitation power of 9.3 mW. The ratios of the three components change only slightly with changing excitation power; at the excitation power of 4.65 mW, the amplitudes of the components at the short wavelength and middle wavelength are 0.31 and 0.60, respectively, while the component at the long wavelength is 0.09. At the excitation power of 9.3 mW, the amplitudes of the components at the short wavelength and middle wavelength are 0.30 and 0.57, respectively, while that of the component at the long wavelength is 0.12. At the excitation power of 4.65 mW, the centre wavelengths of the three components are 656.6 nm, 646.6 nm and 634.2 nm, respectively. However, at the higher excitation power of 9.3 mW, the three centre wavelengths are nearly the same as those at excitation power of 4.65 mW, at 654 nm, 647 nm and 635 nm. The variations in the spectral widths with excitation power were similar to that of the centre wavelength (Fig. S3d). The average spectral width of the short-wavelength component is 22.75 nm, which is much broader than the width of 12.59 nm of the middle-wavelength component and of 10.17 nm of the long-wavelength component.

The most direct approach to understanding blinking is to investigate photo-induced loss (surface recombination) in the PL spectrum from a single QD. Unfortunately, the spectrum from a single QD is usually too weak to be measured. However, one possible way to circumvent this problem is to study the spectrum of a single QD on a PhC, provided that the background signal of the PhC slab is weaker than the single-photon level of the QD. Therefore, attempts to decrease the background emission of PhC materials, such as SiN and TiO_2_, are important, as in the study by Kaji *et al.*^4^.

**Figure S4 Comparison of the spectra under one-photon excited fluorescence and two-photon-excited fluorescence.** **a,** Spectra of QDs on blank SiN materials; **b,** spectra of QDs on a SiN photonic crystal with a lattice constant a=520 nm.

On the blank SiN material without PhCs, the normalised spectra of the QDs under different excitation wavelengths were nearly constant (Figure S4a), while they varied on the PhCs (Figure S4b). The centre wavelength of the TPF spectra at an excitation wavelength of 800 nm shifted to the red compared with those of the one-photon excited spectra, and the spectra at shorter wavelengths were more intense.

**Figure S5** **Two-photon excited spectra from QDs centred at 585 nm on a SiN photonic crystal under different excitation wavelengths**, where the lattice constant of PhC is 1000 nm, the excitation wavelength varied from 802 to 760.5 nm, and the excitation power was 9.58 mW. The labels represent the excitation wavelength.

Two-photon excited spectra from QDs centred at 585 nm on a SiN photonic crystal with a lattice constant of 1000 nm under different excitation wavelengths at a constant excitation power of 9.58 mW are shown in Figure S5. Under longer excitation wavelengths ranging from 802.5-786.7 nm, the PL intensities are higher and the centre wavelengths are shorter than those of the shorter excitation wavelengths from 781.3 to 760.5 nm.

**References**

1. Fujita M., Takahashi S., Tanaka Y., Asano T., Noda S. Stimulated inhibition and redistribution of spontaneous light emission in photonic crystals. *Science* **308**, 1296 (2005).
2. Marceddu M., Saba M., Quochi F., Lai A., Huang J., Talapin D. V., Mura A. and Bongiovanni G. Charged excitons, Auger recombination and optical gain in CdSe/CdS nanocrystals. *Nanotechnology* **23**, 015301(2012).
3. Bae W. K., Padiha L. A., Park Y. S., McDaniel H., Robel I., Pietryga J. M. and Victor I. K. Controlled alloying of the core-shell interface in CdSe/CdS quantum dots for suppression of Auger recombination. *Acs Nano* **7**, 3411-3419(2013).
4. Kaji, T. *et al.* Controlled Spontaneous Emission of Single Molecules in a Two-Dimensional Photonic Band Gap. *J. Am. Chem. Soc.* **135,** 106−109(2013).
